# Temperature Effects on the Magnetic Properties of Silicon-Steel Sheets Using Standardized Toroidal Frame

**DOI:** 10.1155/2014/975051

**Published:** 2014-11-26

**Authors:** Cheng-Ju Wu, Shih-Yu Lin, Shang-Chin Chou, Chia-Yun Tsai, Jia-Yush Yen

**Affiliations:** ^1^Department of Mechanical Engineering, National Taiwan University, No. 1, Sec. 4, Roosevelt Road, Taipei 106, Taiwan; ^2^Department of Mechanical Engineering, Hwa Hsia University of Technology, No. 111 Gongzhuan Road, Zhonghe District, New Taipei City 235, Taiwan

## Abstract

This study designed a detachable and standardized toroidal test frame to measure the electromagnetic characteristic of toroidal laminated silicon steel specimens. The purpose of the design was to provide the measurements with standardized and controlled environment. The device also can withstand high temperatures (25–300°C) for short time period to allow high temperature tests. The accompanying driving circuit facilitates testing for high frequency (50–5,000 Hz) and high magnetic flux (0.2–1.8 T) conditions and produces both sinusoidal and nonsinusoidal test waveforms. The thickness of the stacked laminated silicon-steel sheets must be 30~31 mm, with an internal diameter of 72 mm and an outer diameter of 90 mm. With the standardized setup, it is possible to carry out tests for toroidal specimen in high temperature and high flux operation. The test results show that there is a tendency of increased iron loss under high temperature operation. The test results with various driving waveforms also provide references to the required consideration in engineering designs.

## 1. Introduction

Recent development in electrical vehicle raised the demand on developing high efficiency motors. Since the electric motors relied on silicon steel to conduct the magnetic flux, it became important to minimize the iron loss associated with the motor operation, and iron loss measurement also received renewed research interests. With the advancement of the laminated silicon steel sheet technology, the iron loss value is usually as low as a few watts per kilogram. The motors often reach higher than ninety percent of efficiency. The improvement on the performance of the silicon steel now depends on very delicate adjustments, and the measurements taken also become highly accurate.

Conventional iron loss measurement uses Epstein frame and is based on the principle of applying a sinusoidal voltage to the primary (or the excitation) coil while measuring the induced voltage (secondary voltage) and the leakage current. Because the voltage waveforms on the secondary side will be distorted in the near saturation conditions, it is necessary to use the original integration formulae to reach the required accuracy [1]. Also, the iron loss characteristics are inferior when the direction of the laminated silicon-steel sheet and its rolling direction are at an angle of 50–60° [2]. The Epstein testing method applies test to material properties in only two directions (0°, the rolling direction; and 90°, the transverse direction), which underestimates the overall iron loss. Besides, the magnetic field in motor applications with toroidal silicon-steel sheet is isotropic, rendering the operating condition very different. Testing on toroidal specimen is more desirable; however, in the case of the toroidal specimen each test sample is provided with its own winding. Due to the experimental nature, these windings are bound to bear significant manufacturing tolerances. Detailed measurement of some very delicate variations may be impossible to differentiate. There are also interests in measuring the silicon steel behavior in some extreme conditions. Mthombeni et al. [3] proposed a new Epstein measuring strategy, in which only four test pieces and a set of coil frames could be used for measurements over a broad frequency range. Takahashi et al. [4] proposed a special ceramic frame for the test piece that could withstand high temperatures.

To address these needs, this paper referred to the ASTM A927/A927M-04 and related references [5–9] by self-designing a detachable standardized toroidal test frame for measuring the iron loss of toroidal laminated silicon-steel sheets and for isolating the winding tolerances for precise characteristic comparison among similar but different samples. The accompanying power circuit can provide the signal for iron losses under sinusoidal waveform excitation for comparison with existing data. The circuit also permits iron losses measurements with unipolar or bipolar PWM voltage waveforms. This study also provides measurements on the hysteresis loops induced under some specific conditions [10–18] in Section 4.

## 2. The Conventional Toroidal Frames

**Figure 1:**
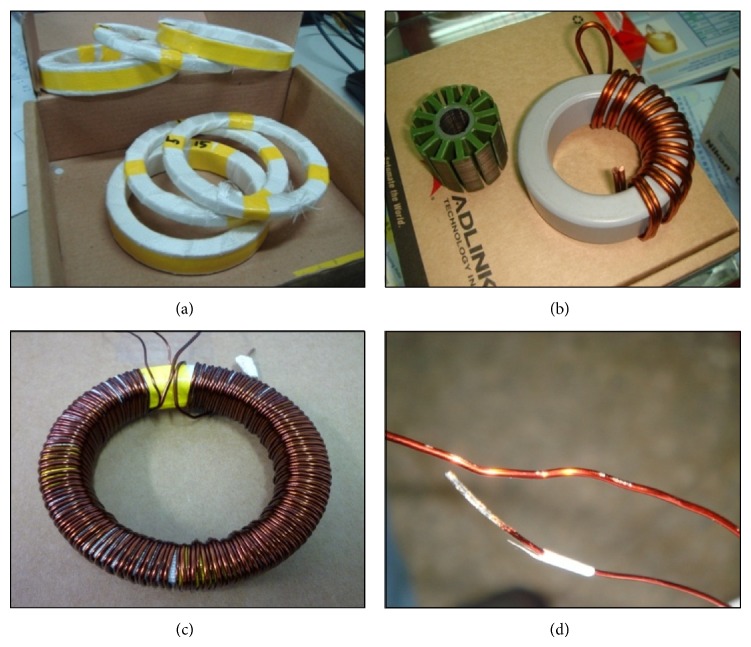
Four subfigures of toroidal laminated silicon-steel sheets used as the control setup in the experiments. (a) Silicon-steel sheets wrapped with glass fiber tape. (b) Sample of a coated toroidal coil. (c) Traditional toroidal transformer used as the control setup in the experiments. (d) Damage to enamel-insulated copper wire that could cause a short circuit.

The preparation of traditional laminated silicon-steel sheets involves stacking the sheets and wrapping the stack with glass fiber tape or coating, as shown in Figures 1(a) and 1(b). For a laminated toroidal silicon steel sheet, the sheets are punched and stack mounted. The process can be detrimental to the material properties of the test specimen and can adversely affect the measured electromagnetic characteristics. Tests carried out on toroidal specimen are therefore desirable.

The toroidal test frame is made the same as a traditional toroidal transformer. There is glass fiber tape to wrap the stack and enamel-insulated wire to wind the primary and secondary coils, as shown in Figure 1(c). The traditional toroidal transformer has various deficiencies, including (1) difficulty in achieving a uniform gap between windings on a laminated silicon-steel sheet, (2) difficulty in winding the coil evenly using an automatic winding machine, and (3) relatively high probability of damaging the surface of enamel-insulated wire to the extent of causing a short circuit and subsequent experiment failure, as shown in Figure 1(d). Furthermore, the traditional toroidal transformer does not allow for the setup of air-flux compensator windings. In general, air-flux compensator windings can be designed using mutual inductance data [2] which may be measured by passing an AC current of 2–5 A through the primary windings of the toroidal frame with no ring specimen in the windings. However, it is not possible to measure the mutual inductance of a toroidal transformer. Thus, there are three major problems when measuring the electromagnetic characteristics of traditional laminated silicon-steel sheets.

1. Inability to rapidly change the toroidal laminated silicon-steel sheets.
2. Inability to design air-flux compensator windings.
3. The differences in the toroidal laminated silicon-steel sheets relative to the original windings.

Since improving upon the deficiencies above would allow the silicon steel sheets to better retain their electromagnetic properties, eliminate winding instability, and shorten time needed for measurement preparations, it is necessary to develop an accurate testing device in which the test piece can be changed rapidly so as to solve the problem of sample preparation as well as to facilitate rapid measurements of various toroidal laminated silicon-steel sheets.

**Figure 2:**
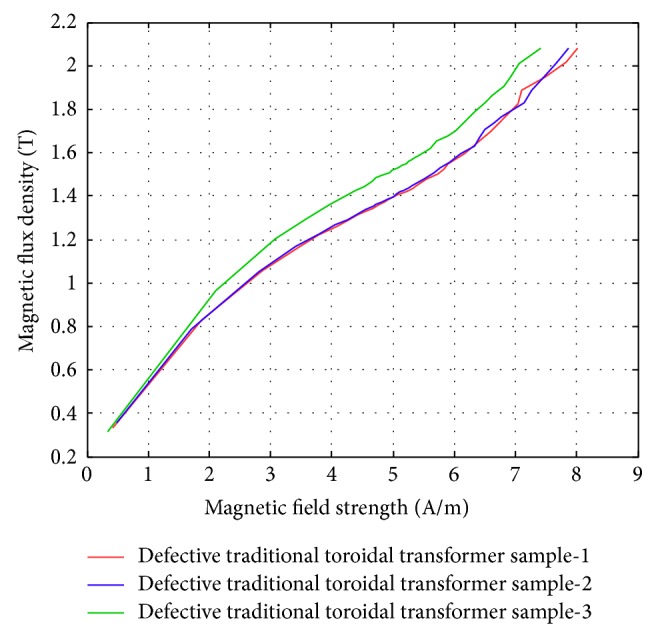
The iron loss curves of the three traditional toroidal transformer samples. The specifications are the same, but the iron loss curves are obviously different (the sample windings are found to be defective).

Figure 2 shows the iron loss curves of three toroidal windings on samples that are of the exact same specifications and have demonstrated significant differences in the iron loss characteristics. Careful examination of the windings does reveal defects in these windings. With the traditional approach, it is unavoidable that each ring sample will have its own winding, unlike the Epstein stand that uses the same coil for all the test sheets. To isolate this factor, we have come up with the idea to design a universal test winding for all the samples.

## 3. Design and Implementation of the New Standardized Toroidal Frame

This section gives more detailed description of the proposed standardized toroidal test frame. As explained before, the standardized toroidal frame is necessary to provide the exact same test environment and to accomplish test flux that exists in all directions.

### 3.1. A New Standardized Toroidal Frame

**Figure 3:**
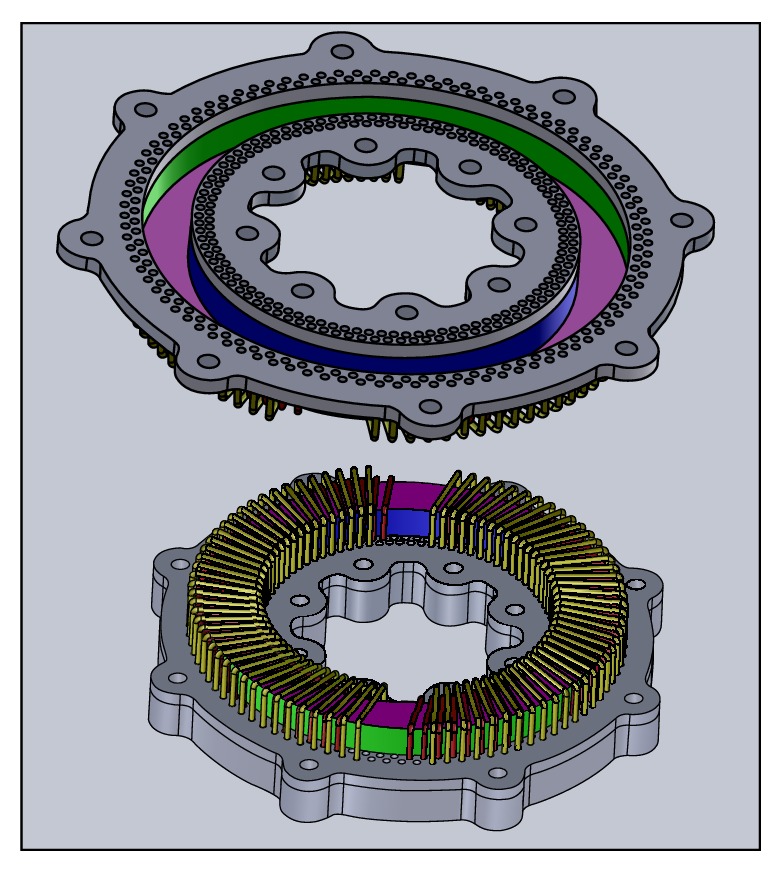
Innovative structure of the new standardized toroidal frame with high repeatability.

This section describes the detailed design of the proposed detachable standardized toroidal test frame for laminated silicon-steel sheets as shown in Figure 3. This test frame is constructed from phenol resin paper and resistant to high temperatures, while the winding form and end pieces may be made from any nonconducting, nonmagnetic material. Nonmagnetic screws and bolts are used in its assembly [2].

**Figure 4:**
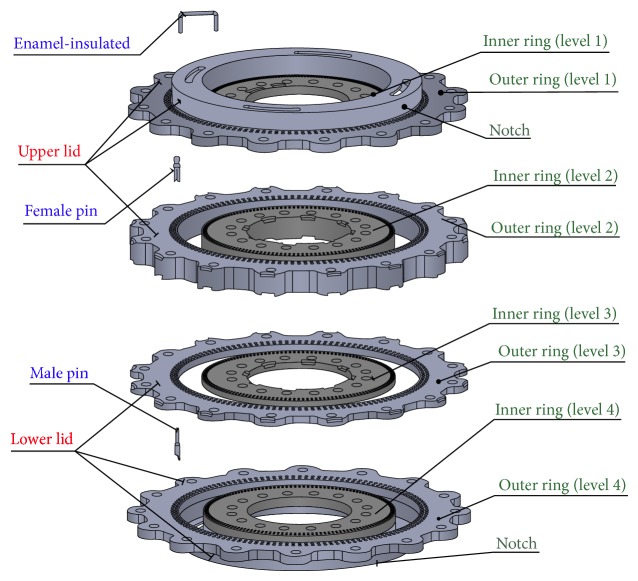
Components of the new toroidal frame.

The upper and lower lids are sliced into two layers each, Levels 1 and 2 for the upper lid and Levels 3 and 4 for the lower lid, so that the toroidal test piece can be placed between the two lids. Each level is then divided into an inner ring and an outer ring, creating a notch in between the two in which to place the toroidal test piece. For levels 1 and 4, the inner ring and outer ring are integrated into single rigid bodies. Thus, the overall design comprises six parts, as shown in Figure 4.

**Figure 5:**
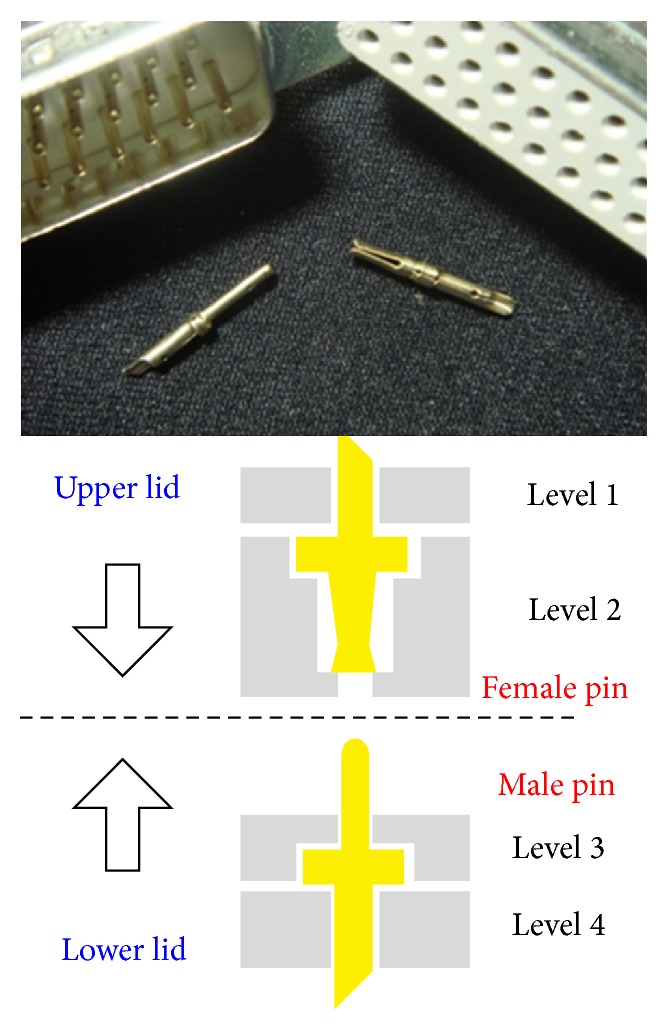
Contact is achieved using D-sub connector solder-type male/female pins.

**Figure 6:**
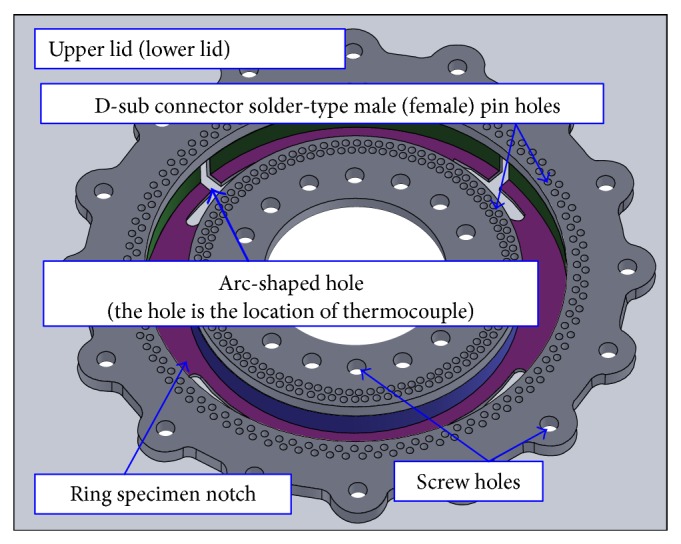
Structure of upper lid (level 1) and lower lid (level 4).

**Figure 7:**
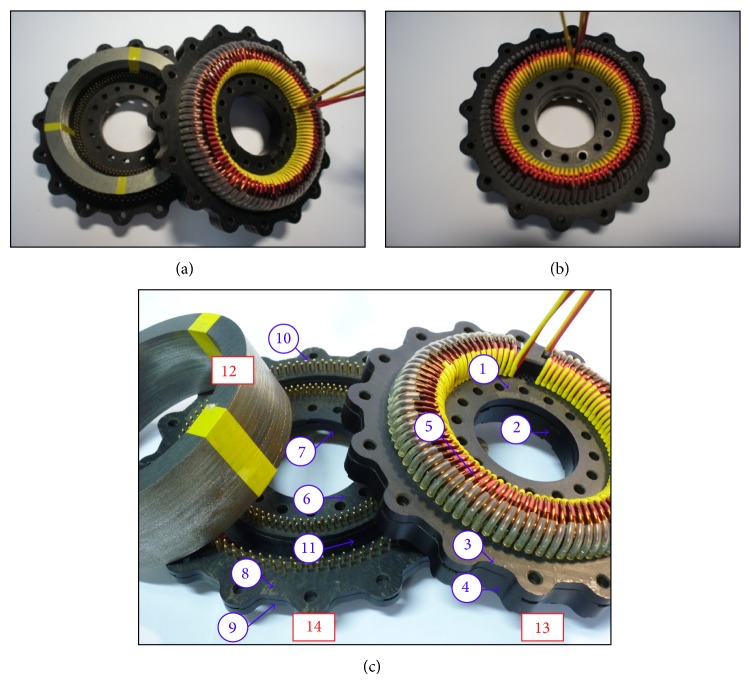
Innovative structure of the new toroidal frame. (a) Standardized toroidal frame and ring specimens. (b) Implementation of the novel structure. (c) Overview of the components of the innovative detachable standardized toroidal test frame.

D-sub connectors inserted into sockets that run through the width of the device are used to connect the coils attached to the outside. Solder-type D-Sub connector pins corresponding to each of the primary and secondary coils are affixed to the base of inner and outer rings of the upper and lower lids: female pins are connected to level 1, while male pins are connected to level 4. There are 90 turns for each of the primary and secondary coils. Enamel-insulated wire is used to connect the inner and outer ring and D-Sub pins, completing the coil; the pin locations are illustrated in Figure 5. Since there are upper and lower lids along with inner and outer rings, the total number of pins is 720. There are also four arc-shaped holes evenly spaced on each of the upper and lower lids, via which the probes or sensor (e.g., temperature detector) can be connected, as shown in Figure 6.  Figure 7(a) shows the innovative structure of the new standardized toroidal frame and ring specimens. The assembled device is shown in Figure 7(b) based on the design specifications listed in Table 1. The device can be easily separated between levels 2 and 3 for easy insertion of the toroidal test piece.

Figure 7(c) shows the overview of this components of the innovative detachable standardized toroidal test frame, including

1. inner ring, level 1,
2. inner ring, level 2,
3. outer ring, level 1,
4. outer ring, level 2,
5. primary and secondary coils,
6. inner ring, level 3,
7. inner ring, level 4,
8. outer ring, level 3,
9. outer ring, level 4,
10. male pin,
11. notch (place the toroidal test piece),
12. laminated silicon-steel sheets,
13. upper lid,
14. lower lid.

### 3.2. Toroidal Test Specimen

The sample laminated silicon-steel sheets have the following specifications: 50A470 material with an outer diameter (*D*_*o*_) of 90 mm, an inner diameter (*D*_*i*_) of 72 mm, and a thickness of 0.5 mm. According to the IEC 60404-6 [19] standard, the *D*_*o*_/*D*_*i*_ ratio of a laminated silicon-steel sheet should not exceed 1.4 and is preferably less than 1.25. The test pieces in our experiment had a *D*_*o*_/*D*_*i*_ ratio of 1.25, which conforms to this standard. The stacked height of the embedded toroidal laminated silicon-steel sheets in the test frame was 30 mm, as indicated in Figure 7 and Table 1. Additionally, the specifications of the traditional transformer used as the control setup were the same as those stated above, with a height of 15 mm, as indicated in Figure 1(c) and Table 2.

### 3.3. Air-Gap Compensation

In order to make more accurate measurements of the electromagnetic characteristics of the laminated silicon-steel sheets, an air-flux compensator winding was connected to the Epstein frame. Since the laminated silicon-steel sheet of a traditional toroidal transformer cannot be removed after winding, it is impossible to construct air-flux compensator windings. However, since the detachable standardized toroidal test frame can be operated with no test piece loaded, our frame design makes it possible to build an air-flux compensator winding when no laminated silicon-steel sheet is present in the toroidal frame. In our experiment, the air-flux compensator windings are designed and constructed according to ASTM A343 [2]. We measured the mutual inductance by passing an AC current of 2–5 A through the primary winding of the test toroidal frame with no ring specimen in the solenoids and the air-flux compensator connected to the coils at the correct polarity. We then used a voltmeter to measure the open-circuit AC voltage at the secondary terminals. If this voltage does not exceed around 2 mV, the air-flux compensator may be assumed to be adequately compensated [2]. The specifications of air-flux compensator windings are listed in Table 1.

### 3.4. Analysis of the Toroidal Electromagnetic Coil

**Figure 8:**
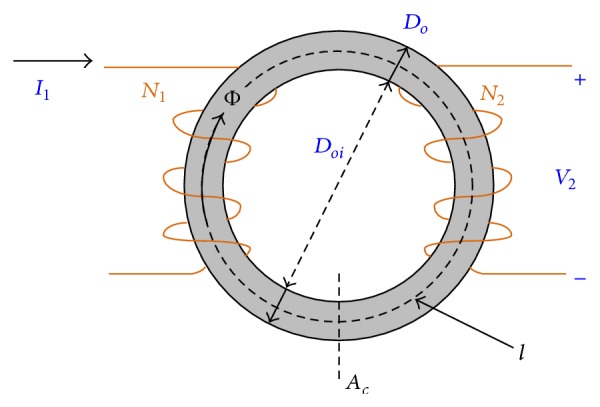
Schematic diagram of the toroidal electromagnetic coil.

The measurement principle of the proposed standardized toroidal test frame is similar to the conventional Epstein test frame. The basic operation of the test is just an open-circuit transformer. The input to the setup is the excitation current to the primary coil, and the magnetic flux induced in the magnetic conductive material causes an electromotive force in the secondary coil. This induced voltage on the secondary coil is then used for the subsequent power loss calculation. As the first step of the calculation, Ampere's law is be used to obtain the magnetic field strength *H*. According to Figure 8, the magnitude of *H* is given by

(1) $$\begin{array}{c} H=\frac{N_{1}\times I_{1}}{l}, \end{array}$$

and Faraday's induction law provides the magnetic flux density *B* as

(2) $$\begin{array}{c} B=\frac{\emptyset }{A_{c}}=\frac{1}{A_{c}N_{2}}\overset{\text{T}}{\underset{0}{\int }}V_{2}dt, \end{array}$$

where *N*_1_ is the number of the primary turns, *I*_1_ is the current through the primary winding, *N*_2_ is the number of the secondary turns, *∅* is the magnetic flux, and *A*_*c*_ is the area perpendicular to magnetic field. Notice that the measured power loss through the primary voltage and current does not represent the iron loss because there is also the primary coil impedance that consumes power. The secondary winding, on the other hand, can serve as a good power indicator. Since the secondary winding is open, there is no current through its terminals. The voltage reflected on the secondary terminal represents the magneto-motive force induced by the magnetic conducting core. Suppose the electrical phase angle between the primary voltage and the primary current is *θ*; then, *I*_1_cos⁡⁡*θ* is the core loss current and *I*_1_sin⁡*θ* serves as the excitation current. This is also the current that is responsible for generating the magnetic flux. As a result, the iron loss as the power *P* consumed by the silicon steel core can be calculated by

(3) $$\begin{array}{c} P=\frac{V_{\text{core}}^{2}}{R_{1}}=V_{2}I_{1}\cos\theta . \end{array}$$

Again *I*_1_ is the primary excitation current, *V*_2_ is secondary induced voltage and cos⁡⁡*θ* is the phase angle.

### 3.5. Method of Measurement and System Architecture

**Figure 9:**
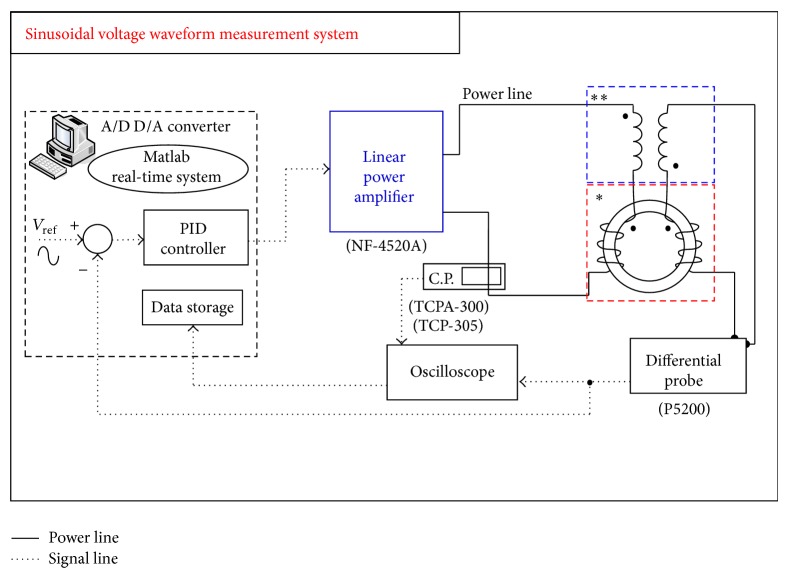
Measurement system under sinusoidal voltage waveform. ^*^Standardized toroidal frame with high repeatability, ^**^air-flux compensator windings.

**Figure 10:**
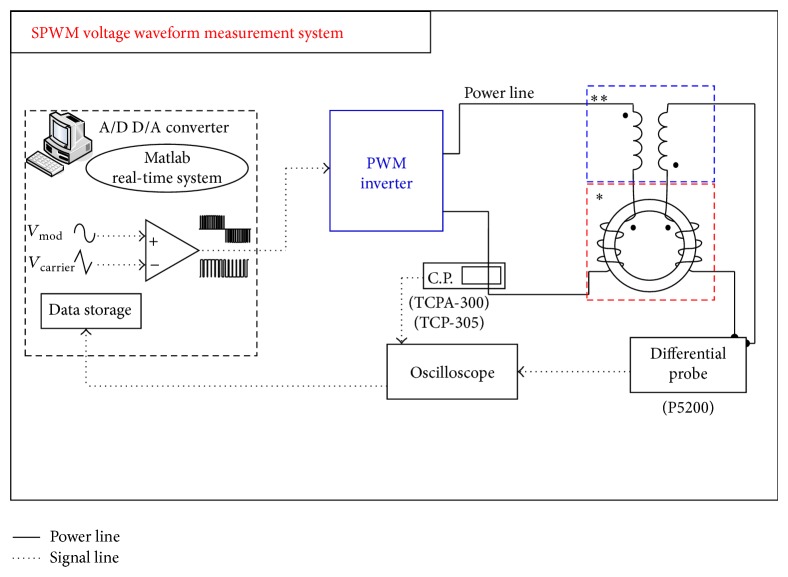
Measurement system under PWM voltage waveform. ^*^Standardized toroidal frame with high repeatability, ^**^air-flux compensator windings.

Figures 9 and 10 show the measurement system setup configuration for sinusoidal voltage waveform excitation and SPWM voltage waveform excitation, where SPWM stands for sinusoidal pulse width modulation. The excitation signals are generated in a MATLAB SIMULINK Real-Time system with NI-PCI-6259 DAQ card interface. A high bandwidth linear power amplifier (with 20 kHz bandwidth) is used to inject the input current. We used the isolated voltage differential probe and current probe to measure the primary excitation current and secondary induced voltage, respectively. A digital oscilloscope is used to monitor the excitation current and the induced voltage. The instantaneous values of the measured excitation current and induced voltage can then be used to calculate the magnetic field strength, the magnetic flux density, and the core loss. The sampling frequency for the measurement system is set at 500 kHz. From Nyquist theorem, the test response could be truthfully reconstructed for frequency components less than 250 kHz. The harmonic components are very small for measurements with pure sinusoidal excitations while their effect becomes significant in the SPWM operations.

There are several variations of the SPWM driving logics. Different driving logic imposes different effects on the induced harmonic components. To truthfully reflect the measurement result, this paper resolves to numerical integration of the measured responses which includes contribution from all the minor hysteresis loops and the high frequency harmonics. We also introduce a current feedback control loop. This is because the excited current can be severely distorted especially at near-saturation regions. As a result the induced secondary voltage (hence the flux) will also become nonsinusoidal. The current loop is then necessary to maintain sinusoidal response [8]. The control loop uses a proportional-integral-derivative (PID) control, as shown the schematics in Figure 9. The measurement results will also include the related waveform form factor to indicate whether the flux density variation is sinusoidal.

**Figure 11:**
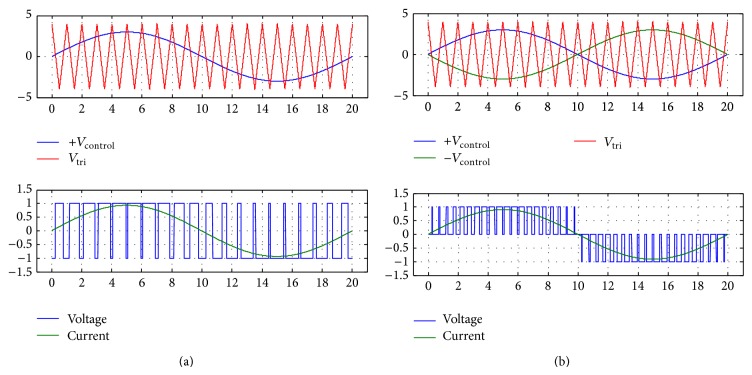
Switching technology (a) Unbipolar Voltage type of PWM—two level Output Voltage, (b) Bipolar Voltage type of PWM—three level Output Voltage.

The second part of the measurement involves the results with the sinusoidal pulse width modulation (SPWM) excitation. The schematics of SPWM experiment setup is shown in Figure 10. The principle of SPWM is shown in Figure 11. By comparing a triangular carrier wave with the sinusoidal signal, it is possible the switch the power on and off to produce a waveform that exhibits sinusoidal power content. The drive can be of unipolar or bipolar waveform as shown in Figure 11. The accompanying circuit in this research is capable of both unipolar PWM and bipolar PWM. There are two parameters/indices to describe the modulation: *m*_*a*_ is the amplitude modulation index; and *m*_*f*_ is the frequency ratio, also known as chopping ratio. The indices are defined as [20]

(4) $$\begin{array}{c} m_{a}=\frac{V_{\sin}}{V_{\text{triang}}},\\ m_{f}=\frac{f_{\sin}}{f_{\text{carrier}}}, \end{array}$$

where *V*_sin_ is the amplitude of the sinusoidal reference voltage and *V*_triang_ is the amplitude of the triangular carrier signal; *f*_sin_ is the frequency of the sinusoidal reference voltage, and *f*_carrier_ is the frequency of the triangular carrier signal.

## 4. Iron Loss Measurement Results

This section presents the main measurement results of the round specimen iron loss under different operation conditions including tests carried for different temperature, frequency, and different modulation indices. One notices that certain operation conditions, although intuitively easier to implement, lead to undesirable high iron losses.

### 4.1. Sinusoidal Voltage Waveform Measurement and Various Temperatures

**Figure 12:**
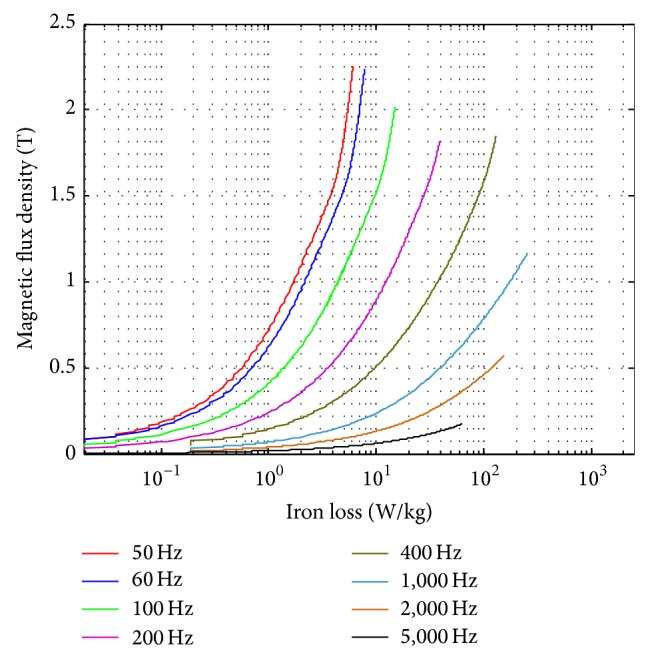
Measurement results obtained from 50 to 5,000 Hz using the new toroidal frame.

**Figure 13:**
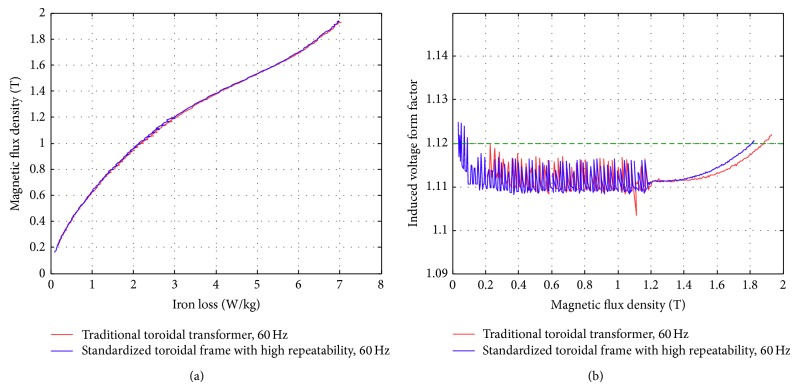
Measurement results obtained at 60 Hz.

**Figure 14:**
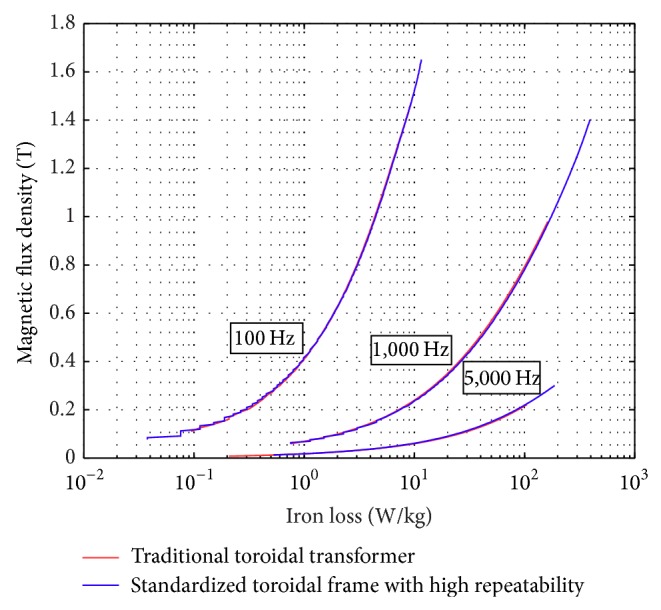
Iron loss measurement results obtained at different frequencies.

Figure 12 shows the iron loss curve for sinusoidal excitation under different frequencies (50–5,000 Hz). The result is as expected with higher iron loss resulted for high frequency operations. It is easier to achieve higher magnetic flux density operating at lower excitation frequencies. Figures 13 and 14 show the comparison of the similar tests conducted on the conventional toroidal transformer frame and on the standardized toroidal frame. It is worth mentioning that the toroidal transformer for this test has been examined with no winding deficiencies, and the tests are carried out on China Steel Co. 50A470 laminated silicon-steel sheet specimen with 60 Hz excitation. According to [2] the iron loss data should be measured for sinusoidal flux variation with form factor within ±1% of $\sqrt{2}\pi /4$ (between 1.01~1.12). This is to guarantee that the test signals maintain good congruency with sinusoidal form.  Figure 13(a) shows the iron loss against varying magnetic flux density. There is a reflection point in the variation and this behavior is similar for both test frames.  Figure 13(b) shows that the form factor of the induced voltage remains within the acceptable range for both frames. Both test frames achieve magnetic flux density as high as 1.9 tesla; however, the conventional toroidal transformer with fixed winding maintains better linearity at higher fluxes.  Figure 14 shows the measurement to reach some extreme flux conditions. The iron loss characteristics maintain the same for both frames.

**Figure 15:**
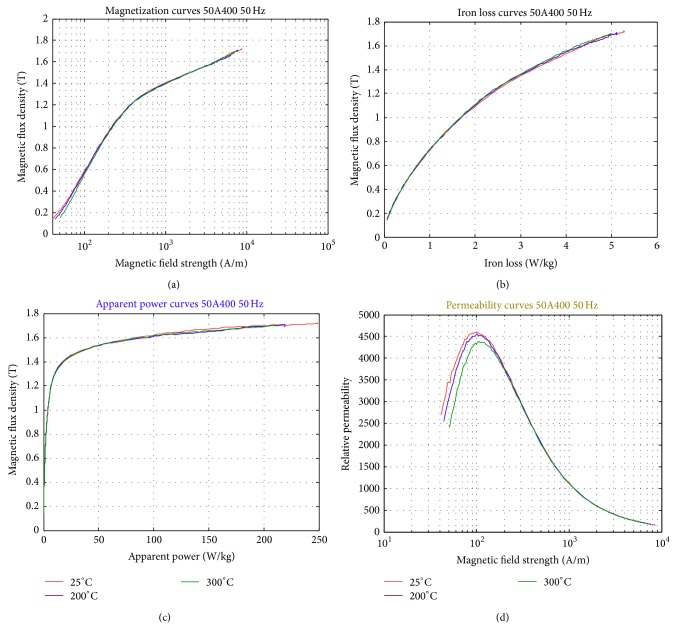
Magnetic properties measurement results obtained at different temperature. (a) Magnetization Curves. (b) Iron loss Curves. (c) Apparent power Curves. (d) Relative permeability curves.

**Figure 16:**
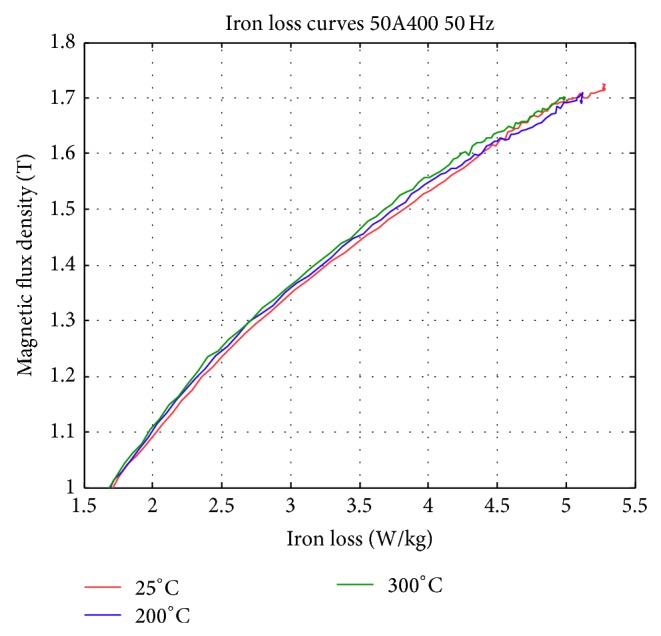
Iron loss partial enlargement measurement results obtained at different temperature. (*B* = 1.0–1.7 T).

The temperature effect measurement is shown in Figures 15(a)–15(d). The temperature range is from 25 to 300°C. One sees that higher magnetic field strength is required at higher temperature to achieve the same magnetic flux, Figure 15(a). Slightly higher flux density is also achieved at lower temperature, Figure 15(b). The power loss, on the other hand, is quite similar for different temperature operations (Figure 15(c)). However, examining the permeability value, one sees that more obvious improvement in permeability is achieved at lower operation temperatures, Figure 15(d).  Figure 16 shows more details in the iron loss curve. Although not clear in the previous figures, it is now clear that lower iron loss is achieved with lower temperature operations.

It is interesting to note that test specimen exhibits lower relative permeability at a higher temperature. This, in turn, leads to lower magnetic field intensity. As a result, the overall iron loss remains the same. Accordingly, the test specimen at a higher temperature will exhibit a lower iron loss with the same magnetic field intensity. This result is also consistent with the temperatures measurements made in [4].

### 4.2. SPWM Voltage Waveform Measurement

This section presents the SPWM iron loss measurements using the standardized toroidal tester. The test specimen were also manufactured from 50A470, and the modulation frequency was 50 Hz. PWM iron loss measurements were made for different values of the amplitude modulation index (*m*_*a*_) and the frequency modulation index (*m*_*f*_) using both unipolar and bipolar switching logics.

**Figure 17:**
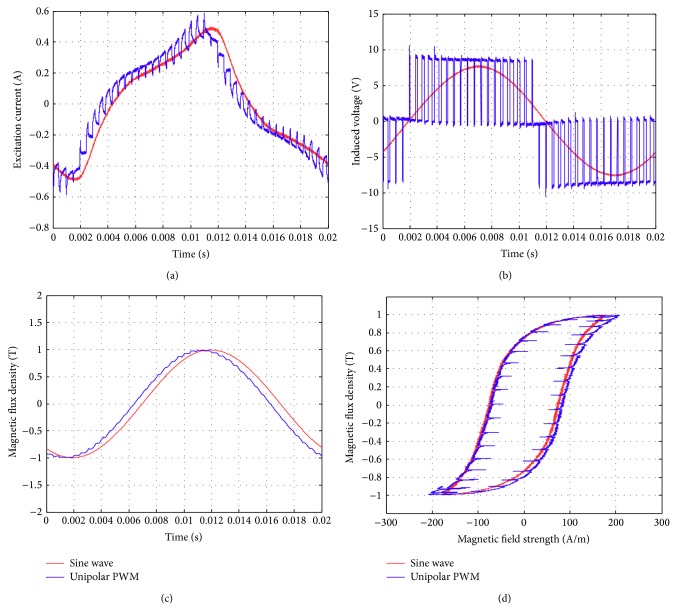
Parameters measured using the new toroidal frame for sinusoidal waveforms and unipolar PWM (with *B* = 1 T, *f* = 50 Hz, *m*_*a*_ = 0.9, and *m*_*f*_ = 20): (a) excitation current, (b) induced voltage, (c) flux density, and (d) hysteresis curve.

**Figure 18:**
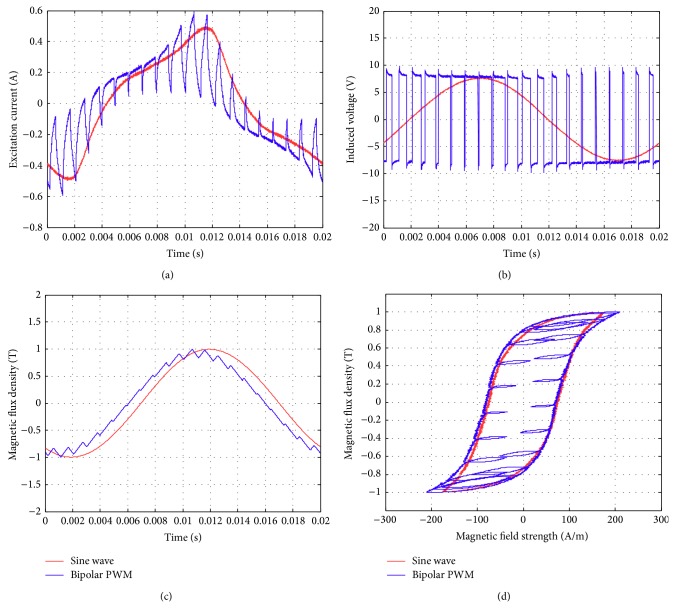
Parameters measured using the new toroidal frame for sinusoidal waveforms and unipolar PWM (with *B* = 1 T, *f* = 50 Hz, *m*_*a*_ = 0.9, and *m*_*f*_ = 20): (a) excitation current, (b) induced voltage, (c) flux density, and (d) hysteresis curve.

**Figure 19:**
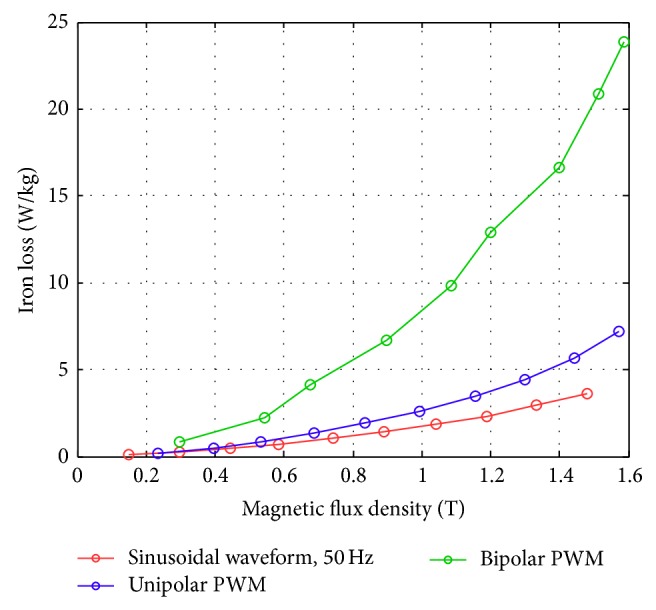
Iron losses for a sinusoidal waveform and PWM (with *m*_*a*_ = 0.3 and *m*_*f*_ = 20) obtained using the new toroidal frame.

**Figure 20:**
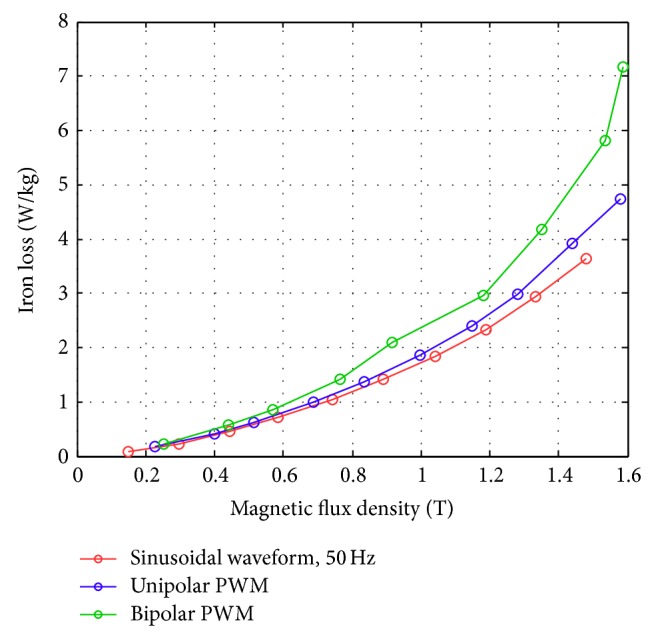
Iron losses for a sinusoidal waveform and PWM (with *m*_*a*_ = 0.9 and *m*_*f*_ = 20) obtained using the new toroidal frame.

Figures 17(a)–17(d) show the comparison of the original sinusoidal waveform and a unipolar PWM experiment. The magnetic flux density in this experiment achieves 1.0 T.  Figure 17(b) shows the unipolar modulation effect, and Figure 17(a) shows how the excited current by PWM input approaches the current from the original sinusoidal excitation. The magnetic flux, however, exhibits almost the same shape as the original sinusoidal signal, Figure 17(c). The magnetization curve in Figure 17(d) now exhibits the effect of minor hysteresis loops due to the PWM effect. The minor loops span a magnetic field strength of around 30 to 50 A/m but contain almost no flux change. Their effect is actually not significant in this test and will become apparent in the bipolar operations. Figures 18(a)–18(d), in turn, show the results from bipolar PWM experiment. The magnetic flux density in this experiment again achieves 1.0 T. Although the basic trend still matches, the excited current differs from the sinusoidal excitation by quite a bit, Figure 18(a).  Figure 18(b) shows the bipolar modulation effect. The signal now oscillates between ±10 volts. The magnetic flux variation still exhibits similar shape as the original sinusoidal signal, Figure 18(c), but there are more apparent oscillations. The magnetization loop in Figure 18(d) now shows large minor loops with some flux changes reaching almost 0.5 tesla. As mentioned before, this study used numerical integration to determine the accurate values for iron loss.  Figure 19 shows that bipolar operation produces excessive iron loss which will be converted into heat and wasted. The unipolar operation, surprisingly, does not generate too much iron loss as one would expect.  Figure 19 shows the results for the tests with amplitude modulation index, *m*_*a*_ = 0.3, and frequency modulation index, *m*_*f*_ = 20.  Figure 20 shows the results for tests with *m*_*a*_ = 0.9 and *m*_*f*_ = 20. One sees that as the amplitude modulation index increases the iron loss also increase, but the difference between unipolar operation and bipolar operation becomes less obvious. Even their difference from the original sinusoidal excitation becomes small. The reduced overall iron loss is an indication that higher amplitude modulation index is more favorable from the energy operation point of view. This will be confirmed with more tests.

**Figure 21:**
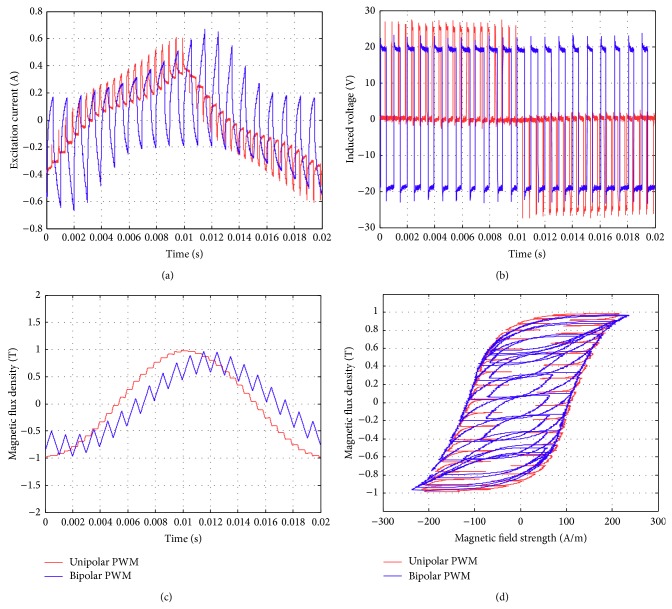
Parameters measured using the new toroidal frame for bipolar and unipolar PWM (with *B* = 1 T, *f* = 50 Hz, *m*_*a*_ = 0.3, and *m*_*f*_ = 20): (a) excitation current, (b) induced voltage, (c) flux density, and (d) hysteresis curve.

**Figure 22:**
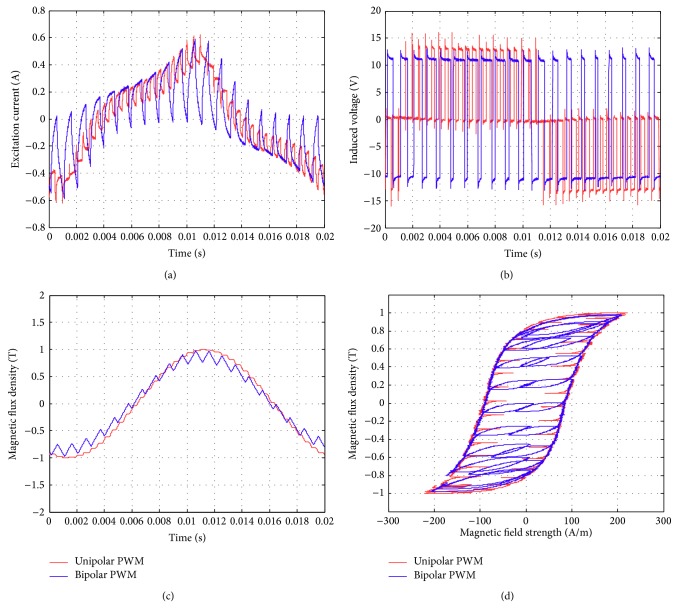
Parameters measured using the new toroidal frame for bipolar and unipolar PWM (with *B* = 1 T, *f* = 50 Hz, *m*_*a*_ = 0.6, and *m*_*f*_ = 20): (a) excitation current, (b) induced voltage, (c) flux density, and (d) hysteresis curve.

**Figure 23:**
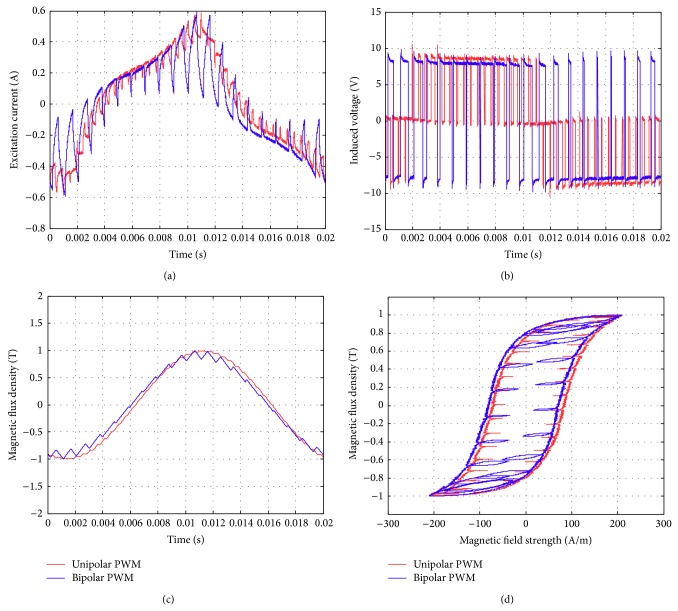
Parameters measured using the new toroidal frame for bipolar and unipolar PWM (with *B* = 1 T, *f* = 50 Hz, *m*_*a*_ = 0.9, and *m*_*f*_ = 20): (a) excitation current, (b) induced voltage, (c) flux density, and (d) hysteresis curve.

Similar tests are now presented in Figures 21, 22, and 23. The subfigures Figures 21(a), 22(a), and 23(a) always show the excited current waveform. Subfigures Figures 21(b), 22(b), and 23(b) show the modulation logic, subfigures Figures 21(c), 22(c), and 23(c) show the flux density variations, and subfigures Figures 21(d), 22(d), and 23(d) show the magnetization diagrams. By comparing these results, it is seen that the minor loops the bipolar PWM are more significant for smaller *m*_*a*_ values. If the iron loss is determined by the calculated hysteresis curve area, the overlapping minor-loop area must be added. As a result, the iron loss is larger for the bipolar PWM than for the unipolar PWM. In addition, there is a clear tendency for decreasing minor hysteresis curve areas and thus decreasing iron loss with increasing *m*_*a*_ values. A summary of the iron losses against various sinusoidal waveforms, unipolar, and bipolar PWM with different *m*_*a*_ values are listed in Tables 3 and 4. These tables indicate that the iron loss was highest for bipolar PWM, followed by unipolar PWM and then the sinusoidal waveform. One concludes that higher *m*_*a*_ value is favorable from the energy point of view, although the resultant energy wave form may be less similar to the original signal and may also tolerate poor resolutions.

**Figure 24:**
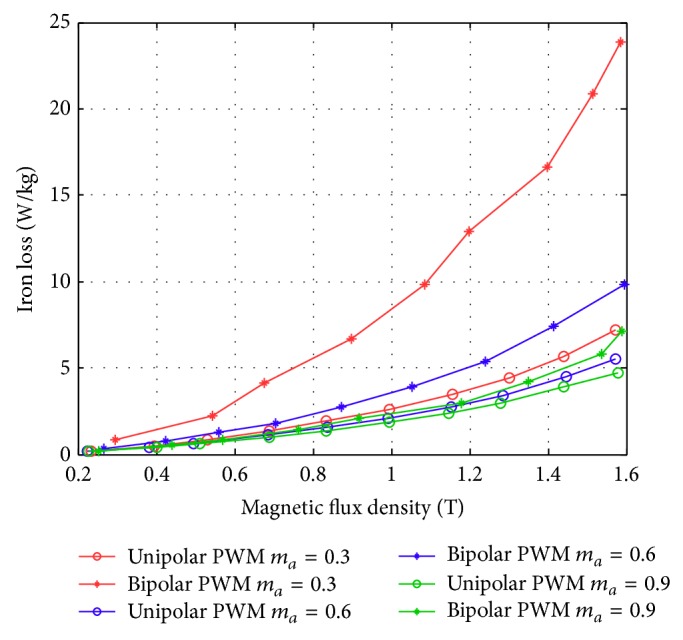
Iron loss versus flux density for bipolar and unipolar PWM (with *f* = 50 Hz and *m*_*f*_ = 20) at different *m*_*a*_ values measured using the new toroidal frame.

**Figure 25:**
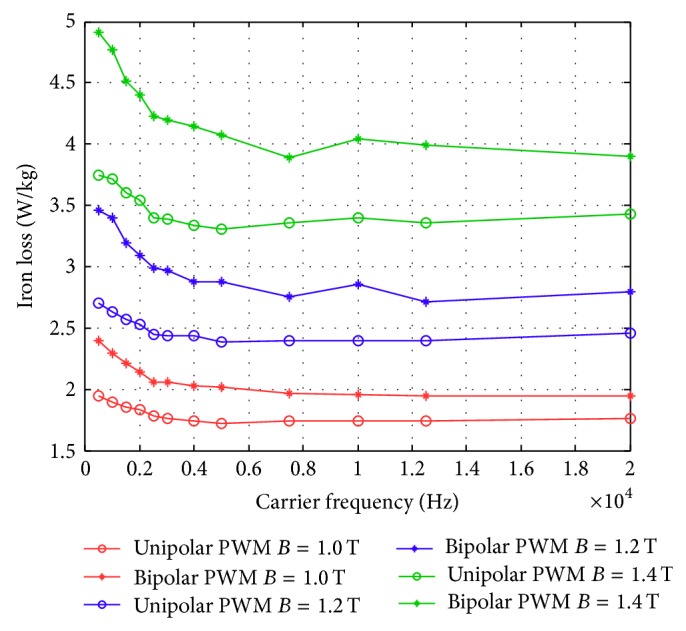
Iron loss versus carrier frequency for bipolar and unipolar PWM at different flux densities measured using the new toroidal frame.

An overall summary figure that combines the unipolar and bipolar PWM is summarized in Figures 24 and 25.  Figure 24 shows that for a modulation frequency of 50 Hz and an *m*_*f*_ of 20; a smaller *m*_*a*_ produces a higher iron loss with the same flux condition. This is consistent with the PWM measurements mentioned in [21]. In addition, the iron loss was higher for the bipolar PWM than for the unipolar PWM due to the larger switching flux density change by the bipolar PWM in the magnetization plots.  Figure 25 shows the test results with *m*_*a*_ set to 0.9. When the carrier frequency was higher than around 8,000 Hz, the iron loss reduces to a constant value while maintaining same flux density. However, the high frequency operation could cause additional losses in the driver circuit. On the other hand, the iron loss was higher for bipolar PWM than for unipolar PWM with the same carrier wave and magnetic flux density.

## 5. Conclusion

An innovative standardized toroidal test frame is proposed in this study to provide precise measurement environment for iron loss tests. The proposed test frame can be used to measure the electromagnetic characteristics of various thicknesses of laminated silicon-steel sheet at high frequencies (50–5,000 Hz) and high magnetic flux densities (0.2–1.8 T). Because the setup withstands high temperatures (25–300°C), it enables iron loss tests for temperature effects. There is an accompanying drive circuit that enables both sinusoidal and nonsinusoidal voltage waveforms. To achieve precision measurement, this study uses numerical integration along the magnetization curve to determine the iron loss values. The measurements lead to some interesting results. The high temperature does not significantly affect the iron loss. Different modulation conditions, on the other hand, can make large difference. Unlike common intuition, higher amplitude modulation condition (higher amplitude modulation index), although achieve less similarity with the original sinusoidal waveform and poor resolution, resulted in less iron loss, and thus is favorable for high energy applications.

## Figures and Tables

**Table 1:** Specifications for the new standardized toroidal frame with high repeatability.

Winding parameters		
	Primary winding	Secondary winding

Solenoid windings	90 turns	90 turns
	1.0-mm AWG #18 wire	0.9-mm AWG #18 wire
Air-Flux Compensator windings	44 turns	8 turns
	1.0-mm AWG #18 wire	0.9-mm AWG #18 wire

Ring specimens 50A470		
Total thickness	Inner diameter	Outer diameter

30 mm	72 mm	90 mm

**Table 2:** Traditional toroidal transformer.

Winding parameters		
	Primary winding	Secondary winding

Solenoid windings	90 turns	90 turns
	1.0-mm AWG #18 wire	0.9-mm AWG #18 wire

Ring specimens 50A470		
Total thickness	Inner diameter	Outer diameter

15 mm	72 mm	90 mm

**Table 3:** Iron losses for a sinusoidal waveform and unipolar PWM (with *f* = 50 Hz) obtained using the new toroidal frame.

Flux density	Unipolar PWM *m*_*a*_ = 0.3, *m*_*f*_ = 20	Unipolar PWM *m*_*a*_ = 0.6, *m*_*f*_ = 20	Unipolar PWM *m*_*a*_ = 0.9, *m*_*f*_ = 20	Sinusoidal waveform 50 Hz
1.0 T	2.66 W/kg	2.21 W/kg	1.94 W/kg	1.71 W/kg
1.4 T	5.46 W/kg	4.31 W/kg	3.80 W/kg	3.25 W/kg

**Table 4:** Iron losses for a sinusoidal waveform and bipolar PWM (with *f* = 50 Hz) obtained using the new toroidal frame.

Flux density	Bipolar PWM *m*_*a*_ = 0.3, *m*_*f*_ = 20	Bipolar PWM *m*_*a*_ = 0.6, *m*_*f*_ = 20	Bipolar PWM *m*_*a*_ = 0.9, *m*_*f*_ = 20	Sinusoidal waveform 50 Hz
1.0 T	7.6702 W/kg	3.4206 W/kg	2.3876 W/kg	1.7192 W/kg
1.4 T	16.6844 W/kg	7.3326 W/kg	4.3311 W/kg	3.2500 W/kg
